# Supplementary material for: Mapping the topographic organization of the human zona incerta using diffusion MRI
Source: bioRxiv. 2026 Jul 10:2024.09.05.610266. Preprint. [Version 2] doi: 10.1101/2024.09.05.610266 (PMC13370454; doi:10.1101/2024.09.05.610266)
Supplement: Supplement 1 [file NIHPP2024.09.05.610266v2-supplement-1.pdf]

bioRxiv preprint doi: https://doi.org/10.1101/2024.09.05.610266; this version posted July 10, 2026. The copyright holder for this preprint (which was not certified by peer review) is the author/funder, who has granted bioRxiv a license to display the preprint in perpetuity. It is made available under a CC-BY 4.0 International license.

Supplementary Figures

Supplementary Figure 1

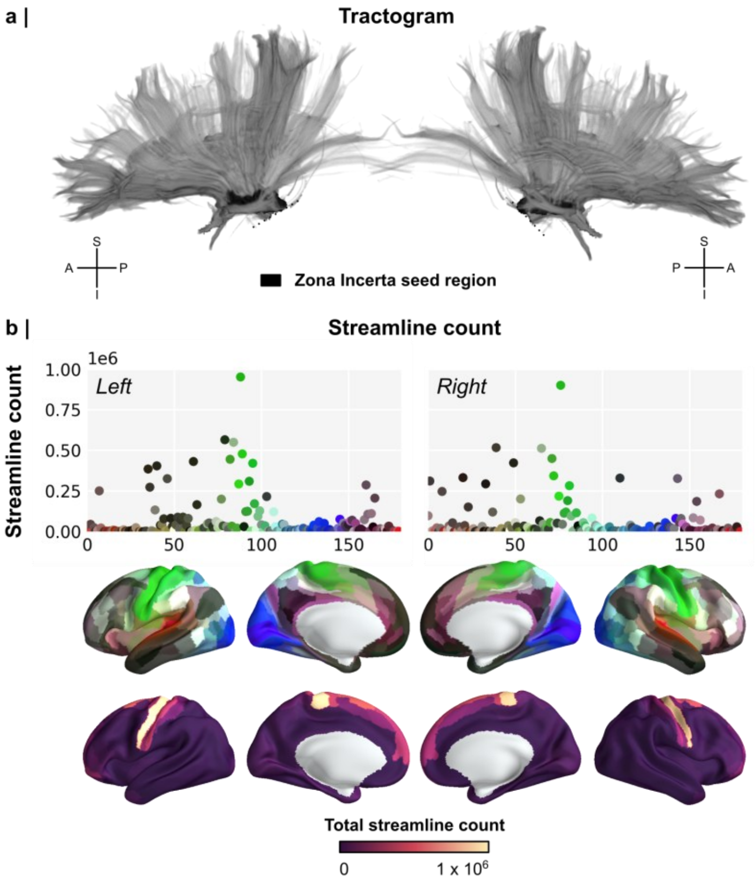

Supplementary Figure 1 - Spectral clustering tractograms and streamline count. (a) Tractogram based on the 7T MRI dataset shown from left vs. right views. (b) Scatter plots showing the streamline count per cortical parcel, color-coded according to the HCP-MMP1.0 atlas as well as a cortical surface representation, color-coded according to the number of streamlines. The source file containing streamline counts is available in the code repository referenced in the manuscript.

bioRxiv preprint doi: https://doi.org/10.1101/2024.09.05.610266; this version posted July 10, 2026. The copyright holder for this preprint (which was not certified by peer review) is the author/funder, who has granted bioRxiv a license to display the preprint in perpetuity. It is made available under a CC-BY 4.0 International license.

Supplementary Figure 2

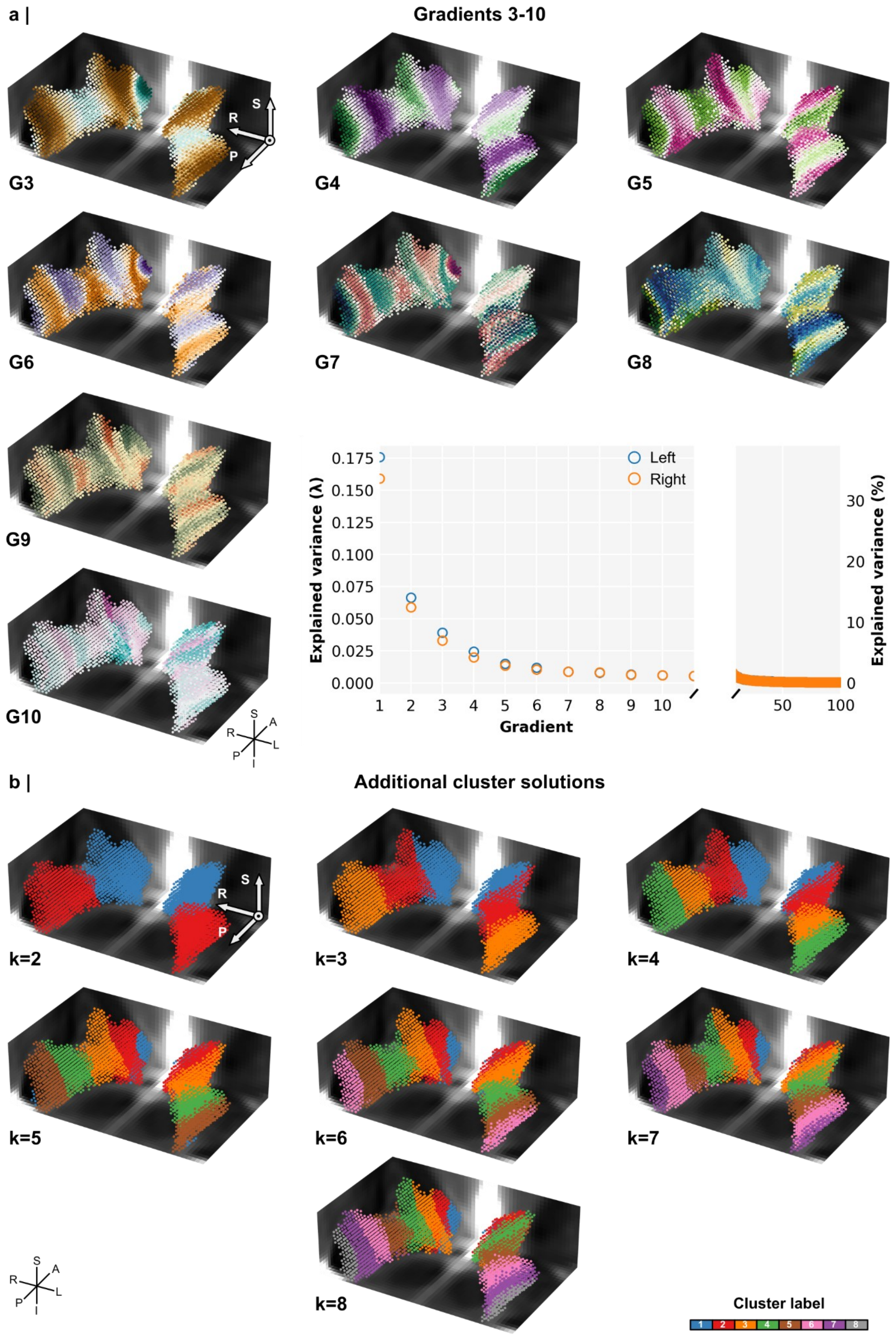

Supplementary Figure 2 - Overview of successive gradients and alternative cluster solutions. (a) Gradients 3-10 (G3-10) shown using 3D radiological views. The scree plot shows the variance explained by each gradient as indicated by their lambda value (left y-axis). The right y-axis displays each gradient's respective contribution (%) to the total explained variance by all gradients. (b) Other spectral clustering solutions (k=2-8).

bioRxiv preprint doi: https://doi.org/10.1101/2024.09.05.610266; this version posted July 10, 2026. The copyright holder for this preprint (which was not certified by peer review) is the author/funder, who has granted bioRxiv a license to display the preprint in perpetuity. It is made available under a CC-BY 4.0 International license.

Supplementary Figure 3

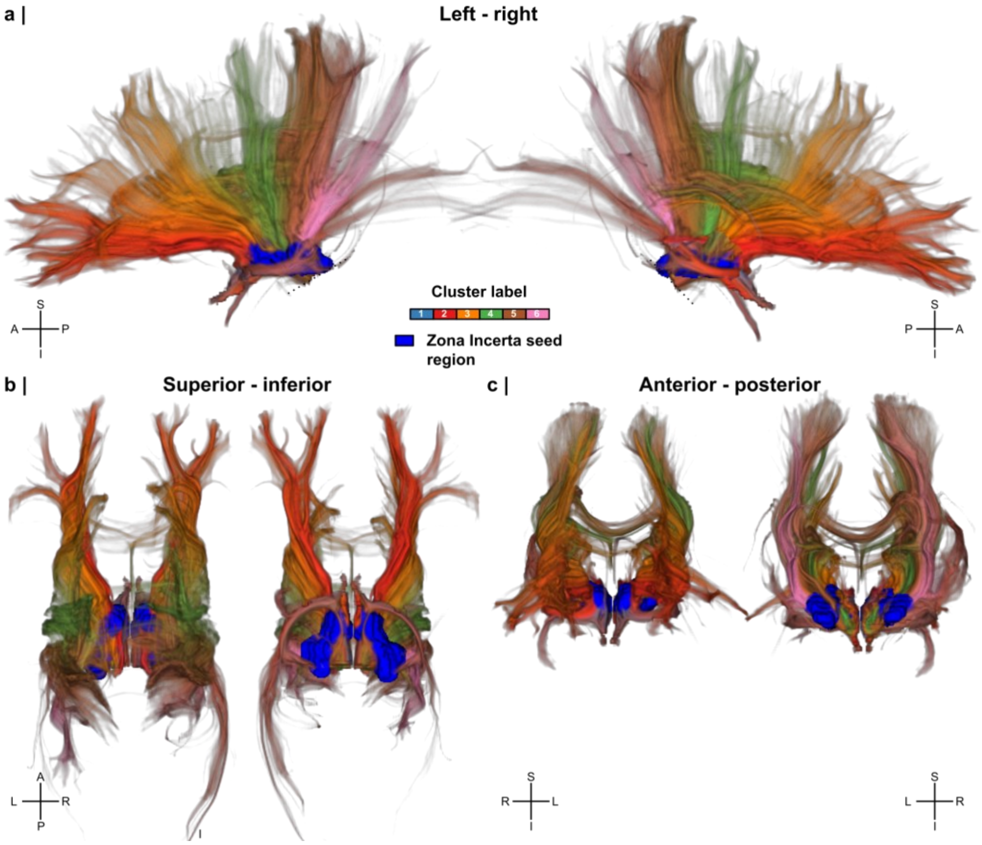

Supplementary Figure 3 - Composite cluster-wise tractogram. (a) Composite tractogram colored by spectral clustering labels, based on the 7T MRI dataset and for k=6 clusters solution, shown from left vs. right, (b) superior vs. inferior and (c) anterior vs. posterior views.

bioRxiv preprint doi: https://doi.org/10.1101/2024.09.05.610266; this version posted July 10, 2026. The copyright holder for this preprint (which was not certified by peer review) is the author/funder, who has granted bioRxiv a license to display the preprint in perpetuity. It is made available under a CC-BY 4.0 International license.

Supplementary Figure 4

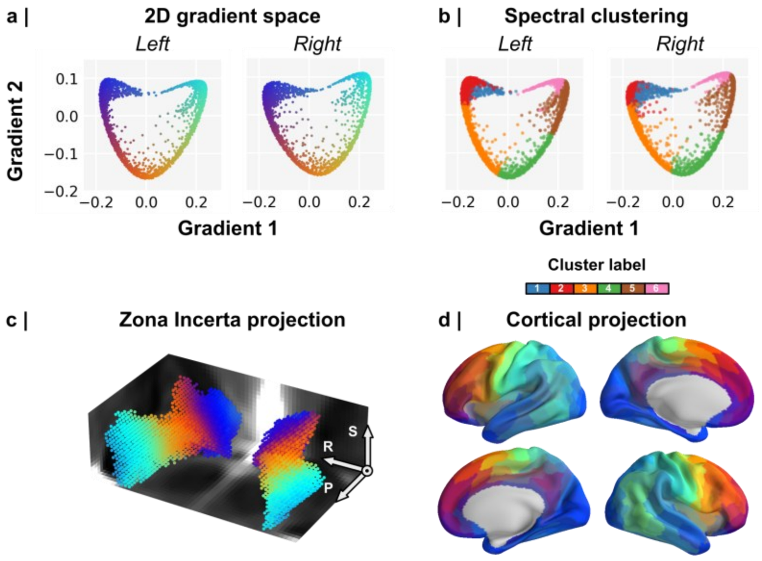

Supplementary Figure 4 - Zona incerta (ZI) 2D gradient coordinate space. (a) Gradient 1 (x-axis) and 2 (y-axis) values were used to position each ZI voxel in the corresponding 2D gradients coordinates space, color-coded using a 2D colormap. (b) Similarly as a, but ZI voxels are color-coded for the k=6 spectral clustering solution. (c-d) Projection of the 2D gradient coordinate space onto the ZI volume and cortical surface spaces, respectively.

bioRxiv preprint doi: https://doi.org/10.1101/2024.09.05.610266; this version posted July 10, 2026. The copyright holder for this preprint (which was not certified by peer review) is the author/funder, who has granted bioRxiv a license to display the preprint in perpetuity. It is made available under a CC-BY 4.0 International license.

Supplementary Figure 5

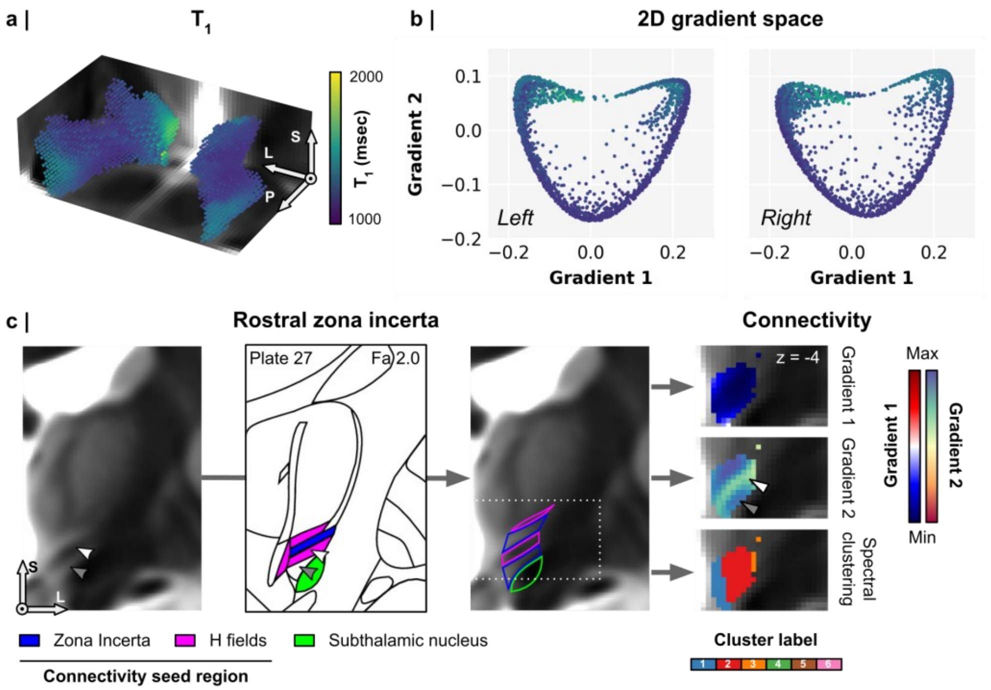

Supplementary Figure 5 - Zona incerta (ZI) longitudinal relaxation times (T_1). (a) 3D volumetric, radiological display of ZI T_1 values (msec). (b) ZI voxels in the 2D gradient coordinate space color-coded for T_1 value. Voxels with high gradient 2 values are characterized by longer T_1 values. (c) Comparison of the rostral ZI T_1, Schaltenbrand atlas, and gradient and spectral clustering results (left to right). Rostral ZI T_1 differences align with changes in gradient 2 values.

bioRxiv preprint doi: https://doi.org/10.1101/2024.09.05.610266; this version posted July 10, 2026. The copyright holder for this preprint (which was not certified by peer review) is the author/funder, who has granted bioRxiv a license to display the preprint in perpetuity. It is made available under a CC-BY 4.0 International license.

Supplementary Figure 6

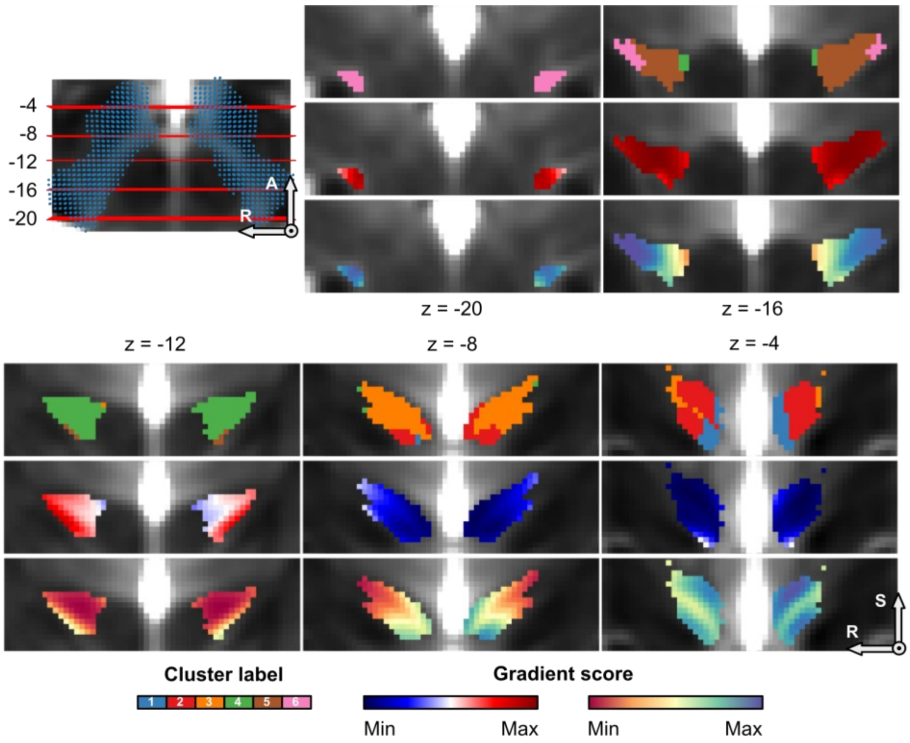

Supplementary Figure 6 - Coronal cross-sections of zona incerta gradient and spectral clustering results.

bioRxiv preprint doi: https://doi.org/10.1101/2024.09.05.610266; this version posted July 10, 2026. The copyright holder for this preprint (which was not certified by peer review) is the author/funder, who has granted bioRxiv a license to display the preprint in perpetuity. It is made available under a CC-BY 4.0 International license.

Supplementary Figure 7

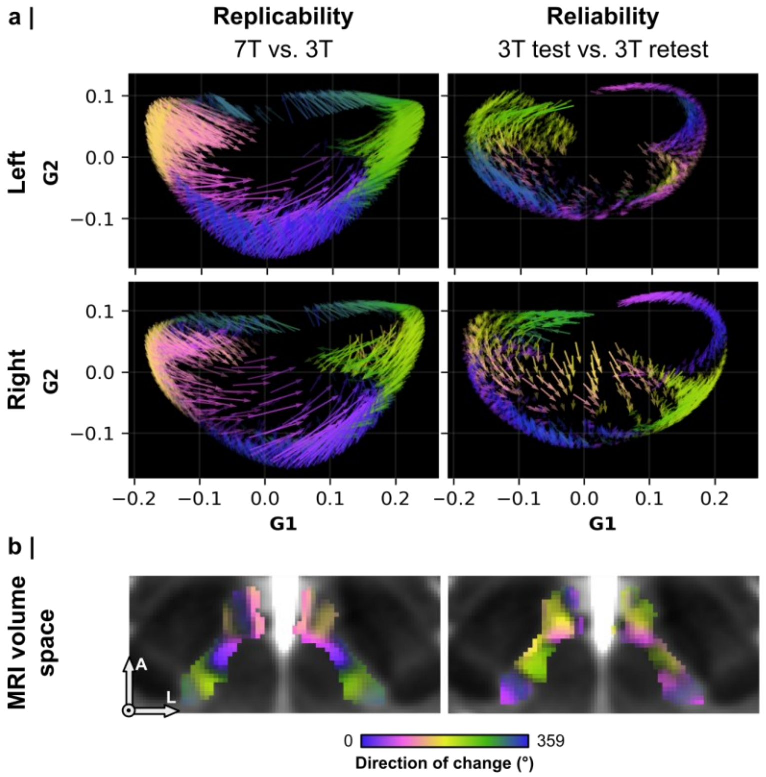

Supplementary Figure 7 - Visual display of the comparison between MRI datasets in the 2D gradient coordinate space. (a) Each arrow in the quiver plots illustrate the shift in gradient 1 (G1) and gradient 2 (G2) values between datasets for a single voxel, color-coded for the direction of change. (b) Projection of the quiver plots into the MRI volume space for localization purposes.

bioRxiv preprint doi: https://doi.org/10.1101/2024.09.05.610266; this version posted July 10, 2026. The copyright holder for this preprint (which was not certified by peer review) is the author/funder, who has granted bioRxiv a license to display the preprint in perpetuity. It is made available under a CC-BY 4.0 International license.

Supplementary Figure 8

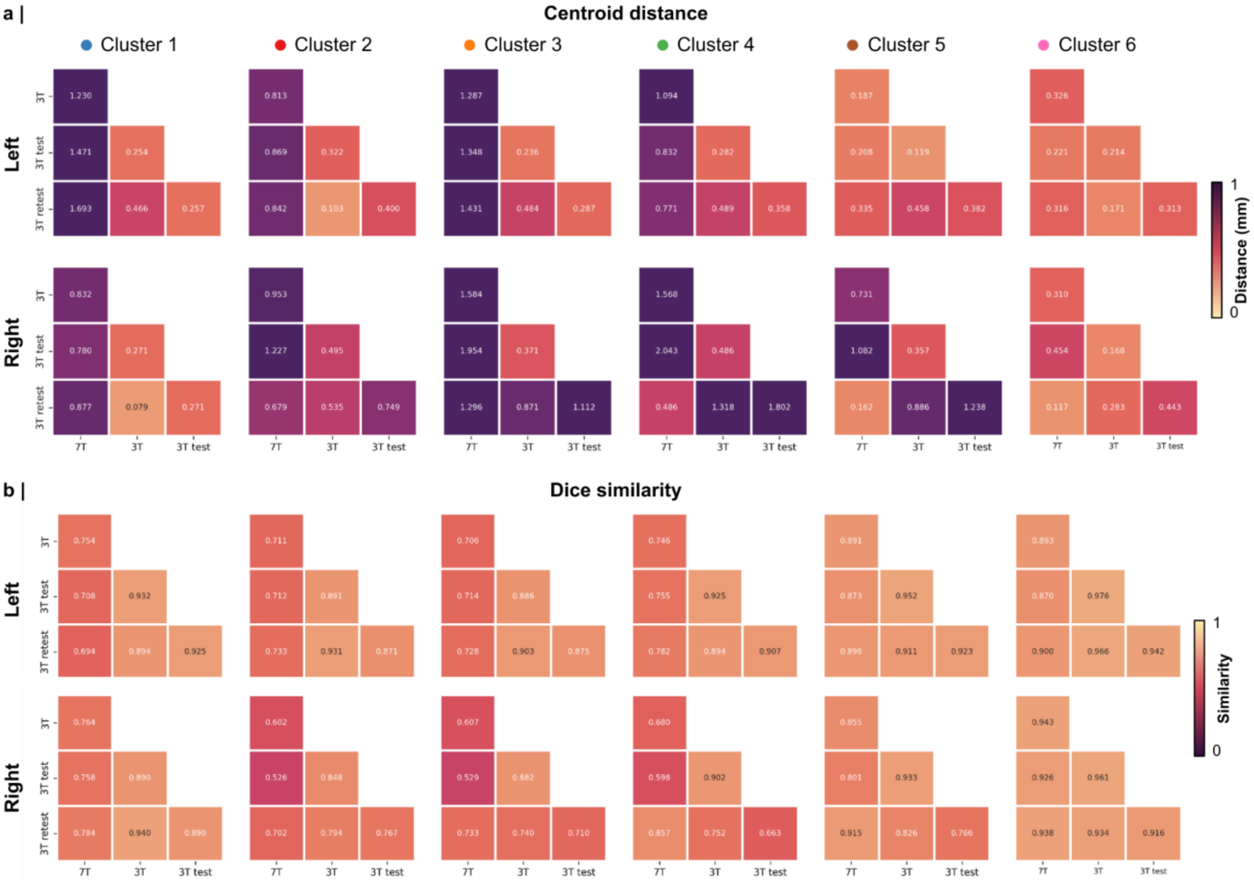

Supplementary Figure 8 - Replicability and reliability of the spectral clustering results. (a) Centroid distance (mm) and (b) Dice similarity scores, split per hemisphere and cluster.

bioRxiv preprint doi: https://doi.org/10.1101/2024.09.05.610266; this version posted July 10, 2026. The copyright holder for this preprint (which was not certified by peer review) is the author/funder, who has granted bioRxiv a license to display the preprint in perpetuity. It is made available under a CC-BY 4.0 International license.

Supplementary Figure 9

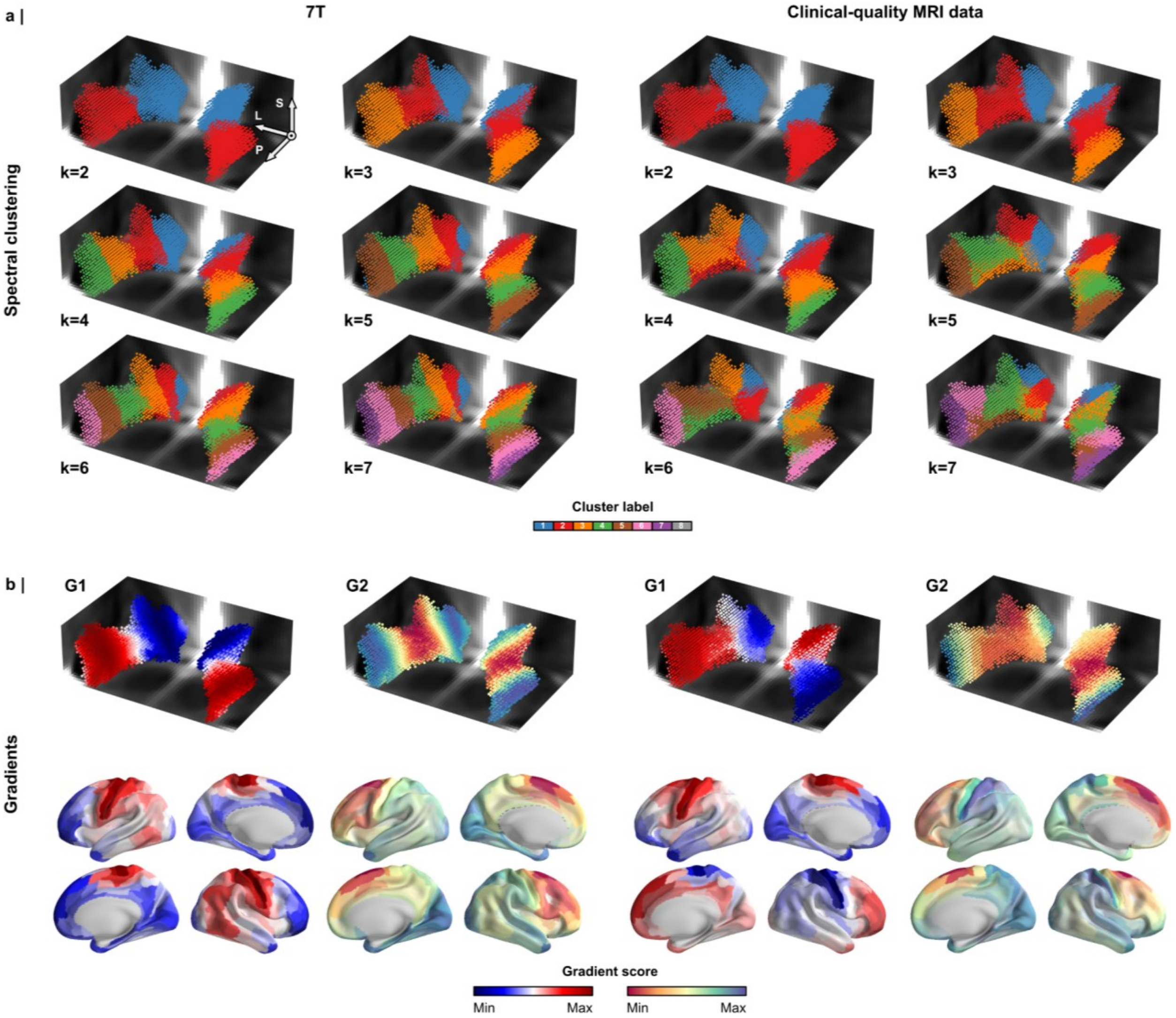

Supplementary Figure 9. Comparison of spectral clustering and connectivity gradients across diffusion MRI acquisition protocols. (a) Spectral clustering results and (b) connectivity gradients obtained from the high-resolution 7T HCP dataset and an independently acquired clinical-quality diffusion MRI dataset (2 mm isotropic resolution with a reduced number of diffusion-encoding directions).

bioRxiv preprint doi: https://doi.org/10.1101/2024.09.05.610266; this version posted July 10, 2026. The copyright holder for this preprint (which was not certified by peer review) is the author/funder, who has granted bioRxiv a license to display the preprint in perpetuity. It is made available under a CC-BY 4.0 International license.

Supplementary Figure 10

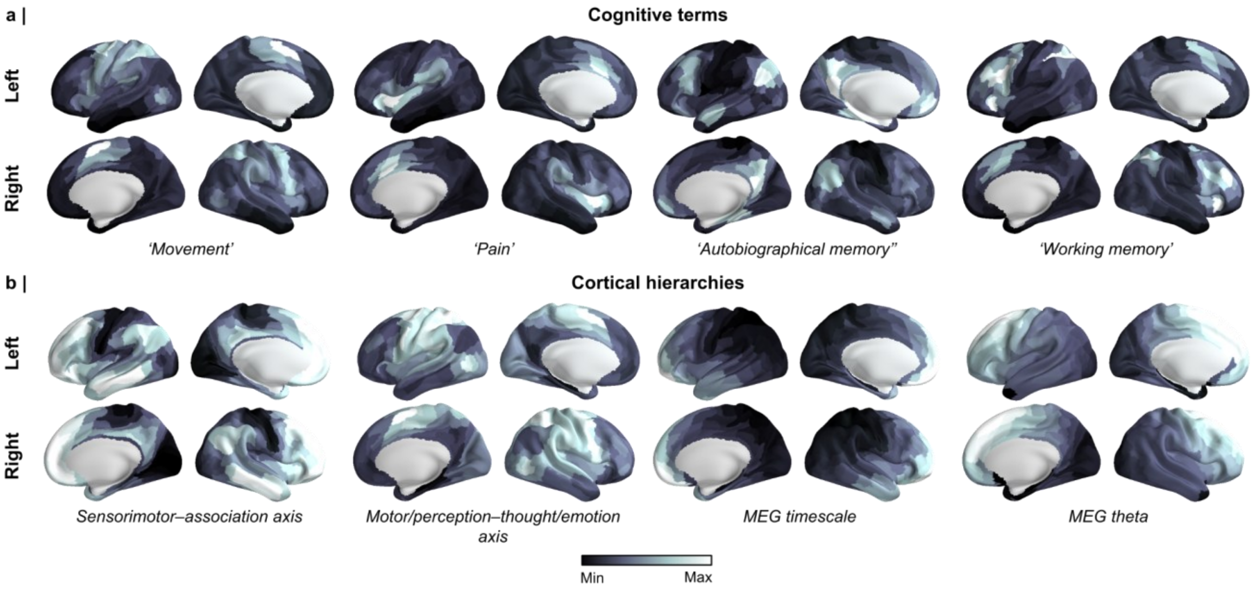

Supplementary Figure 10 - Example cortical (a) NeuroSynth (N=124) and (b) neuromaps (N=73) maps used for contextual analysis. Each cortical map is scaled according to their minimum and maximum value.

Supplementary Table 1 - Top 5% of connections between each zona incerta (ZI) cluster (k=6) and cortical regions. Cortical regions from the left and right hemispheres (HCP-MMP1.0) with streamline counts exceeding the 95th percentile for each cluster are ranked in descending order. The source files used for generating these rankings are available in the code repository referenced in the manuscript.

		**Cluster 1**		**Cluster 2**		**Cluster 3**		**Cluster 4**		**Cluster 5**		**Cluster 6**	
		HCP-MMP	*Brodmann*	*HCP-MMP*	*Brodmann*	*HCP-MMP*	*Brodmann*	*HCP-MMP*	*Brodmann*	*HCP-MMP*	*Brodmann*	*HCP-MMP*	*Brodmann*
**Left**	**1**	PeEc	Temporal	10d	BA10	8BL	BA08	6ma	BA06	4	BA04	1	BA01
	**2**	TGd	Temporal	a10p	BA10	SFL	BA06	SFL	BA06	6mp	BA06	3b	BA03
	**3**	10d	BA10	a47r	BA47	9m	BA09	6mp	BA06	3b	BA03	4	BA04
	**4**	Pir	Temporal	10pp	BA10	8BM	BA08	4	BA04	1	BA01	2	BA02
	**5**	a10p	BA10	9m	BA09	10d	BA10	SCEF	BA06	3a	BA03	3a	BA03
	**6**	V1	V1	p10p	BA10	9a	BA09	6d	BA06	6d	BA06	7AL	BA07
	**7**	PreS	Temporal	9a	BA09	a47r	BA47	8BL	BA08	6ma	BA06	7Am	BA07
	**8**	a47r	BA47	11l	BA11	6ma	BA06	6a	BA06	5m	BA06	5L	BA05
	**9**	10pp	BA10	8BL	BA08	9p	BA09	8BM	BA08	2	BA02	5m	BA05

		**Cluster 1**		**Cluster 2**		**Cluster 3**		**Cluster 4**		**Cluster 5**		**Cluster 6**	
		HCP-MMP	*Brodmann*	*HCP-MMP*	*Brodmann*	*HCP-MMP*	*Brodmann*	*HCP-MMP*	*Brodmann*	*HCP-MMP*	*Brodmann*	*HCP-MMP*	*Brodmann*
**Right**	**1**	10pp	BA10	10pp	BA10	8BL	BA08	6ma	BA06	4	BA04	4	BA04
	**2**	a10p	BA10	a10p	BA10	SFL	BA06	SFL	BA06	6mp	BA06	3b	BA03
	**3**	V1	V1	10d	BA10	9m	BA09	6mp	BA06	3b	BA03	1	BA01
	**4**	TGd	Temporal	9a	BA09	9a	BA09	4	BA04	3a	BA03	3a	BA03
	**5**	PeEc	Temporal	9m	BA09	8BM	BA08	SCEF	BA06	1	BA01	2	BA02
	**6**	10d	BA10	a47r	BA47	6ma	BA06	8BL	BA08	6ma	BA06	5L	BA05
	**7**	8BL	BA08	p10p	BA10	10d	BA10	6d	BA06	6d	BA06	7AL	BA07
	**8**	9m	BA09	8BL	BA08	a47r	BA47	8BM	BA08	SFL	BA06	5m	BA05
	**9**	9a	BA09	11l	BA11	a10p	BA10	s6-8	BA06/BA08	SCEF	BA06	7Am	BA07
